# On the History of the Decline and Final Extinction of Leprosy as an Endemic Disease in the British Islands

**Published:** 1896-06

**Authors:** 


### Reviews of Books.

**On the History of the Decline and Final Extinction of Leprosy as an Endemic Disease in the British Islands.** By GEORGE NEWMAN, M.D. Pp. 149. London: Adlard and Son. 1895.

We have been much interested in this, which is No. 1 of the Prize Essays in connection with the National Leprosy Fund. Its perusal will remove from the mind of the reader the feeling that the active existence of leprosy in these islands has been much exaggerated, as the facts brought together by Dr. Newman will convince him that the incidence of this dire disease upon our ancestors was very extensive. No doubt many other skin affections were included under the term, but that cases of true leprosy — Elephantiasis Græcorum — were but too numerous there is undoubted evidence to show.

Dr. (afterwards Sir) J. Y. Simpson, in his *Edinburgh Medical and Surgical Journal* articles in 1841–42, to which Dr. Newman owns his indebtedness, showed that there is no ground for the belief that the disease was imported into this country by the Crusaders, as there is good proof that it was here as long ago as 60 B.C., and that prior to the Crusades at least three leper hospitals were in existence at Canterbury, Northampton, and Chatham. In 959, during the reign of Edgar, a law was passed making leprosy a valid ground for divorce. There must have been, at a moderate computation, not fewer than fifty leper hospitals established in this country before the end of the twelfth century, and they were supported by endowments, or alms, or fairs, or collections of money in the markets. It is of local interest to note that, according to Dugdale, "John Earl of Moreton, afterward King of England, gave a croft without Lacford-gate, upon the road to Bath, to certain poor lepers, whereupon was built an Hospital to the honour of St. John Baptist."[1] This, of course, was well outside the city. According to Dugdale there was about the same time also at Lawford's Gate another hospital, St. Laurence's. In William Wyrcestre's time there was, "on the west side of Radcliffe-hill," a leper hospital named after St. Mary Magdalene.[2] Dr. Newman refers to the leper hospitals of Bath, Exeter, Gloucester, Plymouth, and many other places in the West of England.

People of eminence had no immunity from the dire disease, for it is related that many members of royal and noble families were its victims. In the thirteenth century it seemed to have reached its height so far as England and Scotland were concerned, and the end of that century and the beginning of the fourteenth gave signs of its marked decline, and this declension extended to most parts of Europe also. Improved hygienic conditions tended to stamp out the disease, whilst the Famine of 1315 and the Black Death of 1349 were contributing elements. The hagioscope, or squint-windows in churches, are some evidence that a certain amount of separation was carried out. Through these "squints," of which Bridgewater furnishes the best example in this neighbourhood, the leper could be a spectator of the religious ceremonies. Dr. Newman says that "other churches possessed a stone slab let into the sill of the window, and so placed that the leper could receive without actual contact with the administrator of the Sacrament." In the time of Henry VIII. leprosy was far too rare to call for

[1] *Monasticon Anglicanum*, ed. 1830, vol. vi., part ii., p. 670.
[2] See Dugdale, *op. cit.*, p. 774.

more than a very few hospitals. In 1778 Gilbert White of Selborne writes: "A Leper is now a rare sight."

Dr. Newman gives two very interesting tables. The first one is divided into two columns, one relating chiefly to leprosy in England, and the other recording mostly the incidence of leprosy in other countries. The second, somewhat fuller than Simpson's similar table, contains a "List of Leper Houses established in the British Islands during the Middle Ages."

In comparing the various passages of the book we were much perplexed by the absence of an index. Dr. Newman's references, too, are provokingly inexact, *e.g.* "Camden Soc., 1863," and "Lysons, p. 348," are not very helpful.

